# A new grading system for plant-available potassium using exhaustive cropping techniques combined with chemical analyses of soils

**DOI:** 10.1038/srep37327

**Published:** 2016-11-23

**Authors:** Ting Li, Huoyan Wang, Zijun Zhou, Xiaoqin Chen, Jianmin Zhou

**Affiliations:** 1 State Key Laboratory of Soil and Sustainable Agriculture, Institute of Soil Science, Chinese Academy of Sciences, Nanjing 210008, China; 2 College of Resources, Sichuan Agricultural University, Chengdu 611130, China

## Abstract

A new grading system for plant-available potassium (K) in soils based on K release rate from soils and plant growth indices was established. In the study, fourteen different agricultural soils from the southern subtropical to the northern temperate zones in China were analyzed by both chemical extraction methods and exhaustive cropping techniques. Based on the change trends in plant growth indices, relative biomass yields of 70% and 50%, K-deficient coefficients of 35 and 22 under conventional exhaustive experiments, and tissue K concentrations of 40 g kg^−1^ and 15 g kg^−1^ under intensive exhaustive experiments were obtained as critical values that represent different change trends. In addition, the extraction method using 0.2 mol L^−1^ sodium tetraphenylboron (NaTPB) suggested soil K release rates of 12 mg kg^−1^ min^−1^ and 0.4 mg kg^−1^ min^−1^ as turning points that illustrated three different release trends. Thus, plant-available K in soils was classified into three categories: high available K, medium available K and low available K, and grading criteria and measurement methods were also proposed. This work has increased our understanding of soil K bioavailability and has direct application in terms of routine assessment of agriculture soils.

The importance of potassium (K) in both plant growth and soil fertility is widely recognized and has a close relation to the long-term sustainability of the soil in which a plant grows^1^,^2^,^3^,^4^. Hence, characterizing the soil K reserve and its availability to plants is important in determining the K supplying capacity of soils. Currently, soil K is understood to exist in four distinct K pools that differ in their accessibility to plant roots, with reversible transfer of K between the pools^2^,^3^,^4^,^5^. The soluble and exchangeable forms are regarded as rapidly available forms of K: they are replenished by non-exchangeable K (NEK) when they are depleted as a result of plant removal and/or leaching^3^,^4^,^6^, and perhaps by large increases in microbial activity^7^,^8^. Wang *et al*.^9^ reported that the amounts of maximum NEK accounted for 21–56% of the total K of the soils tested. NEK in soil is bound coulombically to negatively charged clay interlayer surface sites and this binding force exceeds the hydration forces between individual K^+^ ions^5^,^10^. The plant availability of NEK depends primarily on the rate at which it can be released as more labile forms (i.e., both exchangeable and soluble)^11^,^12^. Some researchers suggest that the more rapidly NEK is released, the more easily it is utilized by plants^11^,^13^,^14^. However, there are currently no assessment methods or theories available for grading NEK bioavailability^9^.

The release of K from layered silicates is a diffusion-controlled reaction under neutral conditions^15^,^16^,^17^,^18^, although both diffusion-controlled reactions and structural decomposition can occur under acid conditions^19^,^20^. Some researchers have described the release of K by equations containing three simultaneous rate terms^21^,^22^,^23^, which can be divided graphically into simultaneous rates of K release from the surface of the soil complex, the weathered periphery, and the micaceous matrix. However, several researchers have defined K release by two simultaneous reactions, relating to the release of external and lattice K, respectively^11^,^18^,^24^,^25^,^26^. Thus, the question arises as to whether the release rate of K in the soil correlates with the grading of plant-available K in that same soil.

Suitable method is a key for developing sound guidelines for evaluating soil K bioavailability. An estimation of rapidly available K by extracting the soil with neutral ammonium acetate, ammonium chloride, calcium chloride, or ammonium fluoride (Mehlich 3) is the most widely used soil test^27^. Measuring plant-available soil K that is released from NEK reserves is difficult because of the complexity of the dynamic equilibrium among the various forms of soil K during crop growth. Nevertheless, various methods have been established to assess the slowly or potentially available K in soils; for example, extraction by 1 M HCl, boiling in 0.5 M or 1 M HNO_3_^28^,^29^,^30^ and sodium tetraphenylboron (NaTPB)^9^,^11^,^31^,^32^,^33^. However, the dilute or concentrated acids extracting methods, on the one hand, may underestimate soil available K supply capacity; on the other hand, extract some structural K which cannot be utilized by plants^20^,^34^. The NaTPB method allows flexibility as a soil test because the extraction (incubation) time can be varied to alter the amount of K release. It can also take both exchangeable and a portion of non-exchangeable K into account and, thus, would appear to be a good choice to use to determine the relation between the release rate levels of K and the grading of plant-available K in soils.

The hypotheses of the present study were: (i) both the amount of K released into the soil solution and its availability to plants are controlled by the soil K release rate; and (ii) a new grading system for soil plant-available K can be established according to the relations between soil K release characteristic, plant K absorption, plant biomass yield, and K concentration in plant. Thus, the objectives of this study were to: (i) grade plant-available K based on the exhaustion of ryegrass (*Loliumperenne* L.); (ii) rank soil K according to its release characteristics by using NaTPB; and (iii) find the gradations of K deliverability from soil K release rate from soils and plant growth indices.

## Results

### Properties of sample soils

The physicochemical properties of the soils studied are presented in Table 1. The results showed that the pH of the soils ranged from 5.42 (WC, Wangcheng, Hunan) to 8.53 (FQ, Fengqiu, Henan). Thus, the soils ranged from highly acidic to alkaline. The cation exchange capacity (CEC) ranged from 5.98 to 36.2 cmol kg^−1^. The organic matter (OM) ranged from 6.52 to 39.2 g kg^−1^. The texture of the soils varied from sandy loam (less clay; i.e., low nutrient-holding capacity) to clay loam (more clay; i.e., medium nutrient-holding capacity). Total K (TK) varied from 16.3 to 28.1 g kg^−1^ with an average of 22.9 mg kg^−1^. HNO_3_ extracted-K varied from 310.6 to 1431.0 mg kg^−1^ with an average of 768.9 mg kg^−1^. The greatest soil NH_4_OAc extracted-K occurred in SHZ (Shihezi, Xinjiang) in the northwestern region of China (319.7 mg kg^−1^), whereas the lowest was in GD (Guangde, Anhui), in the middle and lower reaches of the Yangtze River (33.6 mg kg^−1^). The clay fractions of soils collected from the northwestern region, loess plateau, and northeastern region were rich in terms of their illite content, followed by kaolinite. Kaolinite is the dominant clay mineral in the soils sited in southern China followed by illite.

### Acquisition of potassium by conventional exhaustive experiment

**Figure 1:**
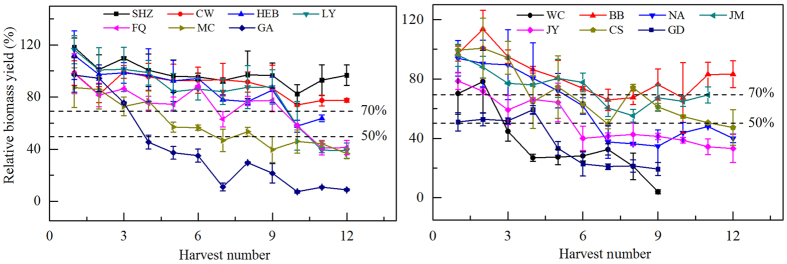
Relative biomass yield for ryegrass at each harvest for 14 soils under conventional exhaustive experiment. Dotted lines in the figures indicate the critical values of relative biomass yield in the current study.

Plant biomass yield is a key factor for describing K bioavailability in soils. As indicated by the relative biomass yield (RBY) from all crops (Fig. 1), the ryegrass only grew well on the SHZ, CW (Changwu, Shanxi), and BB (Beibei, Chongqing) soils, with the mean RBY of 12 crops grown during the study varying from 82% to 98%, followed by ryegrass grown in soils in HEB, LY, and JM, with values from 74% to 82%. On soils in FQ, MC (Mengcheng, Anhui), NA (Nanan, Chongqing), JY (Jiangyan, Jiangsu), and CS (Changshu, Jiangsu), the growth of ryegrass was seriously stunted and the mean RBY varied from 51% to 72%. Ryegrass died before the ninth harvest in WC and GD soils because of K deficiency, so there are no data for any subsequent harvests.

The RBY during the growth period decreased slightly if its values were >70% (Fig. 1), but decreased substantially when its values varied from 50% to 70%. However, when the values of RBY were <50%, the curves began to flatten. Thus, RBYs of 70% and 50% were determined to be critical values in the current study.

Values of RBY, tissue K concentration (Kc), relative K concentration (RKc), and relative K uptake (RKu) for ryegrass were occasionally erratic because of differences in the physical characteristics of the soil, which affected the drainage and aeration in the pots^7^. Principal component analysis (PCA) was applied to evaluate the K-deficient coefficient of ryegrass under a conventional exhaustive experiment by considering all the growth indices (eigenvalues >1). The results showed that 85% of the total variance was explained by the first principal components (PCs) (Table 2). The weights of these indicators were assigned based on the percent variance explained by the particular PC. For correlated variables, the weights were divided equally; thus, 3.400 eigenvalues from PC-1 were divided among RBY, Kc, RKc, and RKu. Equation 1 explains the PCA-based K-deficient coefficient index:


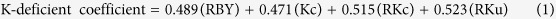


The weight values were normalized to a 0–1 scale by dividing each weighted factor by the total weighted factor (∑wi, 1.998; Equation 2).


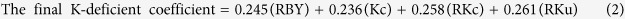


**Figure 2:**
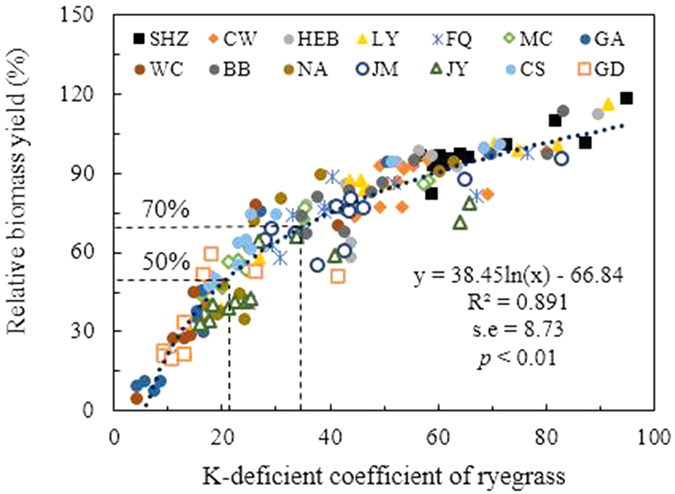
Relation between relative biomass yield and K-deficient coefficient of ryegrass under conventional exhaustive experiment. The right-angled lines in the figures reflect the inflexion points of K-deficient coefficient of ryegrass based on the critical values of relative biomass yield.

We established the relation between RBY and the K-deficient coefficient of ryegrass under a conventional exhaustive experiment (Fig. 2). A statistically significant correlation (*p* < 0.01) showed that RBY decreased with a decreasing K-deficient coefficient of ryegrass, indicating that K deficiency was the most important growth-limiting factor in our exhaustive experiment. A logarithm equation was found to best describe the relation between RBY and the K-deficient coefficient of ryegrass (R^2^ = 0.891) (Fig. 2). The critical values of the RBY (70% and 50%) were used to grade the K status in soils to judge available K supplication to plant growth. According to these parameters, the ryegrass K-deficient coefficients of 35 and 22 were obtained as inflexion points in the current study.

### Acquisition of potassium by intensive exhaustive experiment

**Figure 3:**
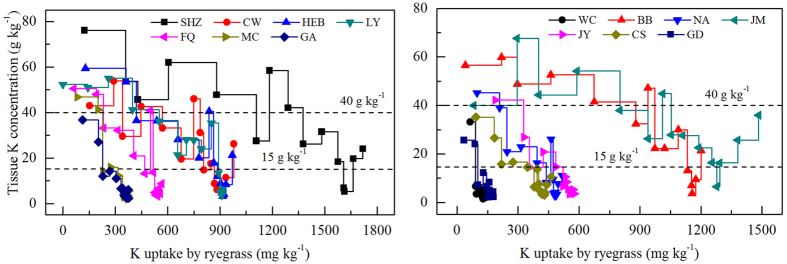
Relation between tissue K concentration and K uptake accumulation of ryegrass under intensive exhaustive experiment. Dotted lines in the figures show the turning points of K concentration of ryegrass based on the change trend of tissue K concentration and K uptake accumulation of ryegrass.

The trends for growth indices of ryegrass grown on different soils under an intensive exhaustive experiment were similar to the trends observed under the conventional exhaustive experiment (Fig. 3). Ryegrass could not survive to produce a 15th crop without external K when it grew on GA, WC, JY and GD soils. The greatest tissue Kc of ryegrass occurred in plants grown in SHZ, CW, HEB, BB, and JM soils for the first to the final harvest, followed by LY, FQ, NA, and CS soils. The cumulative K uptake at the 15th or 14th harvest ranged from 140.5 mg kg^−1^ to 1719 mg kg^−1^ with a mean value of 747.6 mg kg^−1^.

Figure 3 shows the relation between tissue Kc and Ku of ryegrass grown in different soils. On average, when the values of Kc were >40 g kg^−1^, the Ku of ryegrass increased rapidly, and varied from 138 mg kg^−1^ to 1358 mg kg^−1^. When the values of Kc varied from 15 g kg^−1^ to 40 g kg^−1^, the Kc of ryegrass decreased quickly, but the amounts of Ku only increased slightly. When the values of Kc were <15 g kg^−1^, the decrease in Kc and increase in Ku were both slow. Thus, we assumed that the tissue Kc of 15 g kg^−1^ and 40 g kg^−1^ were as turning points in the intensive exhaustive experiments.

### Soil plant-available potassium extracted by NaTPB

To obtain appropriate amount of soil K for predicting soil plant-available K, weak and strong extraction methods were used. For the strong extraction method, it extracted higher amount of soil K than by the weak extraction method and only could use to predict the total amount of plant-available K in soils. Thus, we did not discuss it in this section for the main purpose at there was to rank the soil plant-available K.

**Figure 4:**
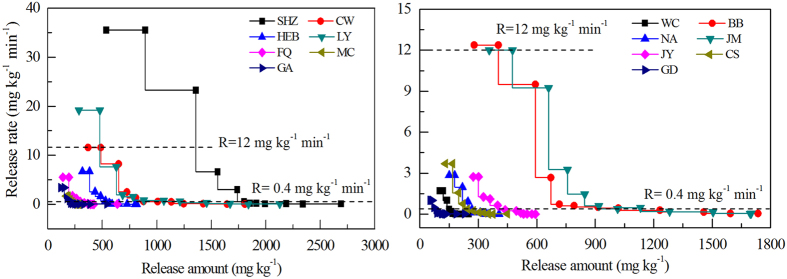
Potassium release amount and rate when extracted by weak extraction method for different soils. Dotted lines in the figures express the critical values of K release rate based on the change trend of K release amount and rate.

For the weak extraction, cumulative K released ranged from 230.7 mg kg^−1^ to 2689 mg kg^−1^, which possibly reflects differences in mineral composition at the different locations^29^. The cumulative released K in soils mainly comprising illite (average of five soils = 1836 mg kg^−1^) was, on average, 3.2 times more than the cumulative K released in soils where the major mineral was chlorite or smectite (Fig. 4). In all soils, the amount of K released after 5-s and 144-h extraction was 1.2–2.2 times and 2.6–15.3 times more than the NH_4_OAc-K released, respectively. In contrast to HNO_3_ extracted-K, NaTPB was found to be least effective in releasing K from kaolinitic soils because the bulk of total K in these samples was present in K feldspars, which are resistant to decomposition by NaTPB^21^. The amount of K released by NaTPB from the sample soils ranged from 1.4% to 9.8% of their total K.

The bioavailability of soil K depends primarily on its release rate and the amount available in the soil^11^,^13^,^14^. To determine the relation between K release amount and rate, the K release rate was plotted against amount to observe the bioavailability of soil K (Fig. 4). On average, when the release rate of K was >12 mg kg^−1^ min^−1^, K was generally released rapidly, and the amount was >400 mg kg^−1^. When the K release rate was <0.4 mg kg^−1^ min^−1^, K was either released slowly or there was no release. Based on this release trend, we classified the soil K into three categories with a release rate of 12 mg kg^−1^ min^−1^ and 0.4 mg kg^−1^ min^−1^ as turning points that represented different release trend: (1) quickly released K, which was rapidly released from the surface of the soil complex; (2) medium released K, which was released from the weathered periphery of the soil complex; and (3) slowly released K, which was released from the micaceous matrix and had the lowest release rate, decreasing successively to zero. SHZ, LY, CW, BB, and JM soils had the highest amounts of quickly, medium and slowly released K, followed by CW, HEB, CS NA, FQ, and JY soils, whereas MC, GA, WC, and GD soils contained the smallest amounts of these three K soil types (Fig. 4).

## Discussion

### Properties of sample soils

The physicochemical and mineralogical property analyses demonstrated that the selected agriculture surface soils represented a wide range of textures with different K status. In fact, the selected soils more or less covered the reported ranges of TK (10–20 g kg^−1^), HNO_3_ extracted-K (200–1600 g kg^−1^) andNH_4_OAc extracted-K (100–400 g kg^−1^) contents of the upper 0.2 m of most agricultural soils^4^,^5^. This was also the original intention of the sample selection, because the underlying aim of the study was to provide a new grading system of plant-available K to optimize the use of the inherent capacity of agricultural soils to sustain long-term K delivery. Hence, it was desirable that the studied soils represented different conditions likely to occur in the field.

### Soil plant-available potassium extracted by exhaustive experiments and by NaTPB

Given that the amount of ryegrass uptake K, the release rate and amount of NaTPB-extracted K represents indexes of K bioavailability under K deficient situations, the soils from SHZ, LY, CW, BB, and JM were hypothesized to release more K more effectively under stress conditions (Fig. 3 and Fig. 4). Similarly, soils from HEB, CS, NA, FQ and JY were hypothesized to release K more effectively under long-term cropping. The lower amounts of ryegrass uptake K and NaTPB-extracted K in soils from MC, GA, WC and GD suggested that these soils would not support enough K nutrition to crops without fertilization under long-term cropping. The lower amounts of ryegrass uptake K and NaTPB-extracted K in these soils could explain by the smaller amounts of illite in the clay mineral compared with the remaining soils^20^,^28^.

### Grading system for soil plant-available potassium

The bioavailability of K in soils was ranked in terms of the potential capacity of a soil to sustain plant growth with no additional K fertilizer, and was mainly related to plant growth and the release characteristics of K in soils^7^,^26^,^33^,^35^. Table 3 shows the available grading criterion of soil K with the parameters we propose. Three categories are detailed in the table: high available K (HAK), medium available K (MAK), and low available K (LAK). In the grading of HAK, the K release rate, RBY, K-deficient coefficient, and Kc of ryegrass were at the highest level and only showed a slight decline. In the rank of MAK, all the parameters significantly declined. In terms of LAK, K was released the most slowly, ryegrass had the lowest RBY (with some unable to grow), and there was the smallest K-deficient coefficient and Kc.

Based on the bioavailable grading criteria for soil K, the relations between the three plant-available soil K categories taken up by ryegrass and extracted by NaTPB were established (Table 4). NaTPB-extracted K showed good linear correlations with plant-available K in the categories of high and medium (*p* < 0.01) bioavailability. In the grading of LAK, a slope of 0.33 indicated that plant-available K under intensive exhaustive experiments was less than NaTPB-extracted K. This trend reflects the fact that soil K levels had become too low to support plant growth, but the low soil K was still extracted by NaTPB, similar to the results of Cox *et al*.^7^. However, the slope and correlation coefficient of the relation between strong NaTPB-extracted K and the cumulative K uptake by plants in the intensive exhaustive experiment showed that 86% of the strong extraction method (NaTPB + NaCl) extracted K during 1 h period was plant available. Thus, it appears that the different plant-available soil K levels can be accurately predicted by the NaTPB method and that the grading criteria for soil K are suitable for ranking plant-available soil K.

A monitoring of soil plant available K is extremely important in order to make precise fertilizer recommendations. Estimations of rapidly and slowly available K by extracting the soil with 1 M neutral ammonium acetate (NH_4_OAc) and boiling in 1 M HNO_3_ are the most widely used soil test^27^. However, plant-available K was well related to NH_4_OAc-extractable K only in soils with low NEK contribution^7^. The extractant by 1 M HNO_3_ is so far not satisfactory for the extraction of plant-available K in soils for at least two reasons. First, it may underestimate soil plant-available K compared to NaTPB^20^. Second, it extract some structural K that is not available to plants^20^,^36^. The grading system based on NaTPB method proposed in this article opens up new prospects for reliable estimates of soil plant-available K because this method can take both exchangeable and a portion of non-exchangeable K into account. The grading criterion presented in this paper is a useful addition to the suite of different K forms tests as it determines the defined fraction of plant-available K in soils. As such this grading system can provide useful information to the planners associated with nutrient management strategy development in gearing up the potassium management.

### Serial measurement methods for the plant-available soil K categories

Predictive ability and convenience in routine work were important considerations in grading the bioavailable K for routine testing^7^. Thus, to quickly and easily obtain amounts for the three plant-available soil K categories, new serial measurement methods for soil bioavailable K are proposed based on Tables 3 and 4. Specifically, the measurement procedures are as follows:

HAK: the amount of K extracted by 10 min 0.2 mol L^−1^ NaTPB subtracting K extracted by 5 s 0.2 mol L^−1^ NaTPB if the value was <120 mg kg^−1^, and the concentration of HAK equal to the amount of K extracted by 5 s 0.2 mol L^−1^ NaTPB. However, if the value was >120 mg kg^−1^, the amount of K extracted by 30 min 0.2 mol L^−1^ NaTPB was considered. If the value of K extracted by 30 min 0.2 mol L^−1^ NaTPB subtracting K extracted by 10 min 0.2 mol L^−1^ NaTPB was <240 mg kg^−1^, then the concentration of HAK was equal to the amount of K extracted by 10 min 0.2 mol L^−1^ NaTPB. By contrast, the concentration of HAK was equal to the amount of K extracted by 30 min 0.2 mol L^−1^ NaTPB.

MAK: if the value of K extracted by 4 h 0.2 mol L^−1^ NaTPB minus the amount of HAK was <92 mg kg^−1^, then the concentration of MAK was equal to the value of K extracted by 4 h 0.2 mol L^−1^ NaTPB subtracting the amount of HAK. By contrast, the concentration of MAK was equal to the value of K extracted by 24 h 0.2 mol L^−1^ NaTPB subtracting the amount of HAK.

LAK: the amount of LAK was equal to the value of K extracted by 1 h 0.2 mol L^−1^ NaTPB + 1.0 mol L^−1^ NaCl subtracting the amount of HAK and MAK.

## Conclusions

A new grading criterion of plant-available K in soils based on the K release rate from soils and plant growth indices was established based on characterizations of soil reserve K and the long-term sustainability of the soil resource. The relation between soil K release amount and rate showed three phases, with release rates of 12 mg kg^−1^ min^−1^ and 0.5 mg kg^−1^ min^−1^ as the cut-off points, based on the extraction method using 0.2 mol L^−1^ NaTPB. In addition, based on the trends in plant growth indices, RBY of 70% and 50%, K-deficient coefficients of 35 and 22 under conventional exhaustive experiments, and tissue Kc of 40 g kg^−1^ and 15 g kg^−1^ under intensive exhaustive experiments were obtained as critical values. Thus, plant-available K in soils was classified into three categories: high available K, medium available K, and low available K. Grading criteria and measurement methods were also proposed. Future research should investigate the utility of this method to budget plant-available reserves of K in different soils.

## Materials and Methods

### Experimental soils

Seven types of arable soils collected from 14 sites spanned from the southern subtropical to the northern temperate zones in China were included in this study (Table 5). The soils were grouped into seven categories based on the geochemistry and climate of the sample site, taken from the agricultural ecological division maps of the sites published by the Institute of Subtropical Agriculture, Chinese Academy of Science: (1) northwestern region [Shihezi, Xinjiang (SHZ)]; (2) loess plateau [Changwu, Shanxi (CW)]; (3) northeastern region [Harbin, Heilongjiang (HEB)]; (4) Huang-Huai-Hai Plain [Laiyang, Shandong (LY);Fengqiu, Henan (FQ); and Mengcheng, Anhui (MC)];(5) Jiangnan region [Gao’an, Jiangxi (GA) and Wangcheng, Hunan (WC)]; (6) Sichuan Basin [Beibei (BB) and Nanan (NA), Chongqing]; and (7) middle and lower reaches of Yangtze River [Jingmen, Hubei (JM); Jiangyan (JY) and Changshu (CS) in Jiangsu; and Guangde in Anhui (GD)].

### Sample collection, preparation, and analysis

Surface soil of 100 kg (0–20 cm) were taken from the plough layer (Ap horizon) during the summer of 2013 after harvest of the crop. Each soil sample was air-dried and ground to pass through a 10-mm or 2-mm sieve; the 10-mm sieved soils (about 60 kg) were prepared for pot exhaustive experiments, and the 2-mm sieved soils (about 1.5 kg) were prepared for soil analysis. The soil pH was determined in a 1:2.5 soil to water suspension. The CEC was obtained by the NH_4_OAc method of Lu^37^ and OM by the Walkley-Black dichromate oxidation method^38^. For determination of the sand, silt, and clay fractions in the samples, the hydrometer method was used^39^. Total K was determined after melting by NaOH^37^. NH_4_OAc extracted-K and HNO_3_ extracted-K were measured by the traditional 1 mol L^−1^ NH_4_OAc method and the 1 mol L^−1^ boiling HNO_3_ methods, respectively^37^. The mineralogy of the clay fraction (<2 μm) was evaluated by X-ray diffraction using an X’Pert-Pro X-ray diffractometer with Cu K α radiation (40 kV, 40 mA) and a graphite filter, from 3.0° to 60.0° with a scan speed of 4.0°/min^20^.

### Acquisition of soil potassium by using crops

As a reference for plant-available K content in soil, conventional and intensive exhaustive experiments both cropping with perennial ryegrass were chosen. Under conventional exhaustive experiment, the soils (5.0 kg) were put in plastic pots 20 cm in diameter and 20 cm deep and arranged in a completely randomized experimental design with three replicates. There were two K treatments: (i) no K fertilizer was applied throughout the experimental period; and (ii) potassium sulfate (200 mg K kg^−1^ soil) was applied as a K fertilizer. To ensure that the general nutrient supply did not limit plant growth, basal nutrients were applied initially and after each harvest^7^,^26^,^35^. The first basal application was given before initiation of the experiment and soils were allowed to equilibrate for 1 week at field capacity; they were watered with deionized water every 1–2 days to maintain soil moisture close to 80% of field capacity throughout the pot culture period. The whole experiment was carried out in a greenhouse with ambient light at a temperature range of 15–35 °C. Ryegrass seeds of 2.5 g were cropped in each pot and the aboveground parts of the plants were harvested after they had grown for 30 d. After harvest, the soil in each pot was thoroughly mixed, the roots of the ryegrass were cut into 0.5–1 cm segments and then returned to the soil before repotting. Ryegrass ultimately died prior to the ninth harvest for WC and GD soils due to K deficiency, and it could not survive to the eleventh harvest of HEB and JM soils due to management misconduct behavior, whereas 12 crops were collected on the other 10 soils.

To enable the rapid removal of K by cropping, an intensive exhaustive experiment was conducted. The soils (5.0 kg) were put in plastic pots measuring 40 cm × 20 cm × 10 cm (length × width × height). Management measures were the same as conventional exhaustive experiment but with no replicates and no K fertilizer. Ryegrass seeds (3.5 g) were cropped in each pot. The aboveground part of the ryegrass (>5 cm in height) was harvested after its length exceeded 20 cm, then harvested again when the length exceeded 20 cm again until the ryegrass could no longer grow in the pot. The experiment was repeated, sowed anew. Ryegrass could not survive for 14 crops without external K when it grew on the soils of GA, WC, JY and GD, whereas 15 crops of ryegrass were collected from the other ten soils.

The biomass of each crop of ryegrass was determined after the leaves were oven dried to a constant weight. Ryegrass leaves were digested with H_2_SO_4_–H_2_O_2_ for K determination^37^. Plant biomass yield (BY) and K concentration (Kc) were used to determine plant K absorption^27^,^40^,^41^. Cumulative K uptake was the summation of plant uptake for each crop harvest^26^.

### Extraction of soil potassium with NaTPB

The procedure followed for the NaTPB method to extract soil K was similar to that described by Cox *et al*.^7^, Li *et al*.^20^ and Wang *et al*.^33^. In order to predict the plant-available K in different soils, we used two types of extraction method: the first was weak (less amount of K was extracted) and the second was strong (more amount of K was extracted). Samples of 0.5 g soil, in triplicate, were weighed into 50-mL centrifuge tubes. For the weak extraction, 3 mL 0.2 mol L^−1^ NaTPB was added and then the tubes were shaken at 200 rpm for each incubation period (5 s, 10 min, 0.5 h, 1 h, 2 h, 4 h, 8 h, 12 h, 24 h, 48 h, 96 h, and 144 h). For the strong extraction method, 3 mL 0.2 mol L^−1^ NaTPB + 1.0 mol L^−1^ NaCl were added to the tubes, which were then shaken at 200 rpm for incubation periods of 1 h. Following the final incubation period in each method, 25 mL quenching solution (0.5 mol L^−1^ NH_4_Cl + 0.14 mol L^−1^ CuCl_2_) was added to the tubes to stop the extraction of soil K. The tubes were then heated in boiling water for 60 min to dissolve the potassium tetraphenylboron (KTPB) precipitate, after which the suspension was vacuum filtered through membrane filters and stabilized by the addition of 1-mL 6 M HCl. The K solution was then measured with a flame photometer (Model HG-5, Beijing detection instrument Ltd.) following an internal standard procedure using 0.003 mol L^−1^ lithium chloride.

### Statistical analyses

All data are the mean of three repetitions (n = 3). Simple linear correlations and nonlinear regressions between variables were calculated using the Linear and Nonlinear Regression functions of SigmaPlot 12.0, respectively. Principal component analysis (PCA) was applied using SPSS software version 20.0 (SPSS Inc., USA) to consider ryegrass growth indices and to confirm their weights to evaluate the K-deficient coefficient of ryegrass under a conventional exhaustive experiment^42^. The principal components extracted from the variables were retained on the basis of the Kaiser criterion of eigenvalues >1.00.

## Additional Information

**How to cite this article**: Li, T. *et al*. A new grading system for plant-available potassium using exhaustive cropping techniques combined with chemical analyses of soils. *Sci. Rep.* **6**, 37327; doi: 10.1038/srep37327 (2016).

**Publisher's note:** Springer Nature remains neutral with regard to jurisdictional claims in published maps and institutional affiliations.

## Figures and Tables

**Table 1:** Basic properties of the soil samples tested.

Site abbreviation	pH	CEC (cmol kg^−1^)	OM (g kg^−1^)	Sand (%)	Silt (%)	Clay (%)	TK (g kg^−1^)	HNO_3_ extracted-K (mg kg^−1^)	NH_4_OAc extracted-K (mg kg^−1^)	Main K-bearing minerals*
SHZ	8.19	20.9	27.7	14.8	61.0	24.2	27.4	1310.8	319.7	I† (24%††), Ch (11%), K (13%)
CW	8.28	13.4	15.6	6.1	76.2	17.7	25.2	1431.0	180.7	I (25%), Ch (21%), K (10%)
HEB	6.84	30.5	38.6	13.2	68.4	18.4	27.2	1175.1	223.6	I (20%), K (17%), Ch (7%)
LY	6.84	6.0	20.9	72.9	19.6	7.5	23.3	972.0	138.9	I (26%), Sm (12%), Ch (8%)
FQ	8.53	6.3	13.6	60.1	28.8	11.1	23.1	681.8	88.7	I (15%), K (15%), Ch (9%)
MC	7.12	10.1	12.5	8.2	76.6	15.2	17.5	542.5	110.1	I (9%), K (2%), Ch (1%)
GA	5.82	17.3	39.2	18.1	60.5	21.4	26.2	829.7	99.5	I (10%), K (8%), Ch (2%)
WC	5.42	7.8	34.9	7.4	58.5	34.1	16.8	310.6	76.9	I (8%), K (4%), Ch (7%)
BB	5.97	36.1	22.8	31.6	48.2	20.2	28.1	655.7	129.6	I (20%), Ch (17%), V (5%)
NA	7.48	20.2	33.7	20.3	47.2	32.5	18.6	357.9	96.0	Ch (15%), I (14%), K (8%)
JM	6.31	36.2	35.5	5.3	59.7	35.0	27.6	1067.6	265.7	Ch (25%), I (19%), V (7%)
JY	7.70	7.7	21.8	44.7	46.4	8.9	20.2	604.6	193.0	Ch (10%), I (7%), V (3%)
CS	7.31	18.0	15.5	8.3	70.0	21.7	22.6	474.2	77.0	I (15%), Ch (10%), Sm (7%)
GD	6.51	6.6	6.52	22.7	64.2	13.1	16.3	351.2	33.6	Sm (11%), K (5%), I (4%)

^†^Abbreviations are: I, illite; Ch, chlorite; K, kaolinite; Sm, smectite; V, vermiculite.

^††^Numbers in the parentheses represent the K-bearing minerals percentages of the clay minerals.

**Table 2:** Results of PCA of the K-deficient coefficient of ryegrass.

Principal components	Eigenvalues	Variance (%)	Cumulative variance (%)	Eigenvectors			
				RBY	Kc	RKc	RKu
PC-1	3.400	84.998	84.998	0.902	0.869	0.950	0.963

**Table 3:** Grading criterion of soil plant-available K.

Plant-available K grading	K release rate in soils by weak extraction method (mg kg^−1^ min^−1^)	RBY of crops (%)	K-deficient coefficient of ryegrass	K concentration of ryegrass under intensive exhaustive experiment (g kg^−1^)
HAK†	>12	>70	>35	>40
MAK	0.4–12	50–70	22–35	15–40
LAK	<0.4	<50	<22	<15

^†^HAK, MAK and LAK are the high available K, medium available K and low available K, respectively.

**Table 4:** Comparison of the amounts of the three plant-available levels that extracted by NaTPB and uptake by ryegrass grown in different soils (mg kg^−1^).

Soil abbreviation	HAK†			MAK		LAK		Total bioavailable K	
	Extraction by NaTPB (y_1_)	Uptake by ryegrass (x_1_)††	Uptake by ryegrass (x_2_)	Extraction by NaTPB (y_2_)	Uptake by ryegrass (x_3_)	Extraction by NaTPB (y_3_)	Uptake by ryegrass (x_4_)	Extraction by NaTPB + NaCl over a 1-h period (y_4_)	Uptake by ryegrass (x_5_)
SHZ	1358	>770	1291	442	428	>348		2149	>1719
CW	486	>462	448	392	400	611	>132	1489	>980
HEB	317	301	361	212	503	541	>107	1070	>971
LY	477	448	398	587	454	131	>64	1195	>916
FQ	138	86	193	159	214	372	>160	668	>566
MC	169	73	202	53	71	192	103	413	376
GA	118	62	113	80	90	242	173	440	377
WC	104	33	64	37		173	76	314	140
BB	403	461	675	511	414	1074	>112	1988	>1201
NA	151	70	98	95	296	566	>123	812	>517
JM	477	392	591	656	664	219	230	1352	1485
JY	275	119	188	125	168	259	220	658	576
CS	132	60	93	91	183	516	>187	739	>463
GD	54	9	34	20	54	226	91	300	179
Correlation		y_1_ = 0.84x_1_ + 86.85 R^2^ = 0.918 p < 0.01	y_1_ = 0.94x_2_ + 13.58 R^2^ = 0.922 p < 0.01	y_2_ = 1.02x_3_ − 45.81 R^2^ = 0.754 p < 0.01		y_3_ = 0.33x_4_ + 169.71 R^2^ = 0.494 p = 0.119		y_4_ = 1.16x_5_ + 104.61 R^2^ = 0.864 p < 0.01	

^†^HAK, MAK and LAK are the high available K, medium available K and low available K, respectively.

^††^Values in the row of x_1_ are the data under conventional exhaustive experiments, and values in the row of x_2_, x_3_, x_4_ and x_5_ are the data under intensive exhaustive experiment.

**Table 5:** Site characteristics of the 14 agricultural sites in China included in the study.

Site	Site abbreviation	Longitude	Latitude	Elevation (m)	Mean annual precipitation (mm)	Mean annual temperature (°C)	Soil type (soil taxonomy)
Shihezi, Xinjiang	SHZ	85°56′43.04′′	44°19′24.34′′	433	225	7.8	Orthicaridosol
Changwu, Shanxi	CW	107°45′27.56′′	35°12′52.61′′	1213	588	9.4	Usticisohumosol
Harbin, Heilongjiang	HEB	126°48′35.00′′	45°51′34.65′′	116	523	4.9	Udicisohumosol
Laiyang, Shandong	LY	120°43′57.73′′	37°0′5.04′′	41	800	12.0	Udiccambosol
Fengqiu, Henan	FQ	114°24′0.06′′	35°00′3.02′′	70	615	14.3	Udiccambosol
Mengcheng, Anhui	MC	116°33′9.67′′	33°9′40.43′′	27	812	15.4	Aquicvertosol
Gaoan, Jiangxi	GA	115°20′59.6′′	28°26′28.61′′	41	1560	18.1	Udicferrosol
Wangcheng, Hunan	WC	112°49′36.8′′	28°16′42.2′′	66	1411	17.7	Stagnicanthrosols
Beibei, Chongqing	BB	106°23′25.98′′	29°47′22.11′′	327	1105	18.2	Udiccambosol
Nanan, Chongqing	NA	106°36′23.28′′	29°31′17.01′′	538	1089	18.0	Udicargosol
Jingmen, Hubei	JM	112°52′31.90′′	30°50′2.77′′	46	1179	16.4	Udiccambosol
Jiangyan, Jiangsu	JY	120°6′1.16′′	32°25′59.86′′	5	992	15.2	Stagnicanthrosols
Changshu, Jiangsu	CS	120°41′57.0′′	31°32′45′′	4	1054	16.2	Stagnicanthrosols
Guangde, Anhui	GD	119°27′18.68′′	31°1′23.46′′	34	1150	15.7	Stagnicanthrosols
